# Virtual health and care: six steps for inclusive policymaking

**DOI:** 10.1186/s44263-024-00050-9

**Published:** 2024-04-01

**Authors:** Ann Aerts, Doreen Bogdan-Martin

**Affiliations:** 1 The Novartis Foundation, Basel, Switzerland; 2 International Telecommunication Union, Geneva, Switzerland

Virtual health and care can expand access to quality healthcare and drive progress towards universal coverage. The Broadband Commission Working Group on Virtual Health and Care proposes a roadmap consisting of six policy pillars to achieve universal access and equity, following the rapid uptake of virtual services due to COVID-19.

## Virtual health: why it matters

The COVID-19 pandemic impacted the delivery of healthcare around the world. As countries looked for solutions to reduce disruptions to critical services, whilst acknowledging social distancing, virtual health and care rapidly became mainstream in many parts of the world, and these services have remained in place to this day.

Following the pandemic, the Broadband Commission for Sustainable Development, a public/private partnership working to help achieve the United Nations development goals through mobile and broadband technologies, launched the Working Group on Virtual Health and Care. The working group was co-chaired by Dr. Tedros Adhanom Ghebreyesus, Director General of the WHO, and Dr. Ann Aerts, Head of the Novartis Foundation, and convened industry leaders, government officials, and civil society to address prominent issues affecting broadband access, affordability, and use. Its focus was to examine the policy levers for the rapid uptake of virtual services in the last few years of the COVID-19 pandemic. It defined “virtual health” as digital solutions that integrate medical, social, and environmental factors to enable holistic well-being, while “virtual care” described solutions that health systems and providers can employ remotely to help patients manage health conditions at a lower cost than traditional care [1]. When combined with in-person services, virtual health and care present a valuable opportunity to expand access to quality, person-centered health services and drive progress toward universal health coverage [1].

In its analysis, the Working Group examined countries’ responses to the increased usage of virtual health services, detailing how many unnecessary restrictions on virtual health and care were swept away in the face of the growing needs of health systems, and how they rapidly incorporated alternative delivery routes. The Working Group provided real-life evidence and usage examples that virtual health and care tools were helping health systems manage widescale challenges with proactive and preventive strategies [2, 3]. For example, telehealth appointments were a common virtual care solution that streamlined communication between patients and providers, conserving providers’ time and resources. “Smart” diagnostics and therapeutics used big data and machine learning to support clinical decisions [1, 4, 5]. Furthermore, real-time digital notifications allowed providers to maintain regular communication with patients to provide timely guidance that streamlined access to care and helped avert disease outbreaks [1, 6].

## Barriers and challenges in virtual health

Despite the growing availability of digital health and care applications, access remains grossly uneven [7, 8]. In 2023, the number of people worldwide not connected to the internet decreased to an estimated 2.6 billion people compared to 2.7 billion in 2022. This leaves 33% of the global population unconnected [9]. As well as a lack of internet access, other communities that may struggle to access digital services include low-income households, people living in rural areas, the elderly, people living with disability, or people with limited digital literacy. This creates a digital divide representing unequal access to virtual health and care tools and applications (i.e., smartphones, tablets, internet) [1]. While there is a clear opening to maximize the opportunities offered by virtual health and care, policymakers need to ensure it is done in a way that builds equitable access. In response, the Broadband Commission report provided a landscape analysis of trends and effects of virtual health and care delivery, highlighting policy actions that promote access and enhance health equity through virtual means. Based on this analysis, the Working Group proposed a roadmap with the detailed policy steps necessary to ensure virtual services help countries address health equity and access challenges.

## Roadmap: a guide to virtual health maturity

In order to achieve universal digital access and equity, countries can follow the Working Group’s proposed roadmap with six policy pillars that offer opportunities to introduce virtual health and care broadly. To make progress, decision-makers must approach each pillar with the intent to create inclusive policies and services.

1. Governance and regulatory systems should maintain inclusive processes for formulating and implementing strategies, plans, and guidelines that underpin equitable access to virtual health and care.
2. Design processes should lead to user-friendly, human-centric solutions developed through an inclusive Research and Development approach removing obstacles to innovation, and designing policies for better health outcomes.
3. Governments should use data and technology to build trust by providing an architecture—information and communications technology infrastructure, data governance, and interoperability standards—where user data are secure and data-use practices abide by the highest privacy standards.
4. Business models should leverage a variety of funding sources, sustainable investment, and innovative pricing schemes to ensure that virtual health and care reaches people across all sections of society.
5. People and workforce policies should enable everyone to be equipped with know-how to access and use virtual health and care services, through training, continuing education, and user support.
6. Decision-makers should create a collaborative, inclusive ecosystem of partners that leads to the wide adoption of virtual health and care solutions [1].

By adopting a virtual health and care maturity roadmap which serves as a guide for health systems and providers to build capacity in these six areas, policymakers can identify the practical steps needed to make virtual health and care accessible to all. Progressing toward maturity in these six areas demands adept leadership, sustained funding, and accountability.

When designed in a human- and equity-centered way, ideally combined with basic healthcare reimbursement schemes that acknowledge people’s socio-economic status, virtual health, and care services have the potential to improve population health by increasing access to and quality of health and care. For example, centralizing expertise can help address the shortage of qualified health professionals, and virtual health appointments can help overcome the geographical, cultural, or other barriers people face to accessing health and care. During the pandemic and its lockdowns, healthcare reimbursement schemes did not include telehealth and virtual health services were sometimes the last resort for patients, especially those with preexisting chronic conditions [10]. This is not reflective of health systems and their utilization post-COVID.

## Conclusion

Policymakers have never faced such a favorable environment for virtual health and care services: the increasing acceptance of virtual technology throughout our lives presents an unprecedented opportunity to harness technology in health and care and accelerate the achievement of Sustainable Development Goal 3 of ensuring healthy lives and promoting well-being for all at all ages. We call on governments, healthcare, and technology stakeholders to leverage this unique momentum and transform health systems into hybrid models, delivering both virtual and in-person health services to everyone who needs it, where they need it.

## Data Availability

No datasets were generated or analysed during the current study.
